# The VASCERN-VASCA diagnostic and management pathways for kaposiform hemangioendothelioma

**DOI:** 10.1007/s00431-025-06631-6

**Published:** 2025-12-13

**Authors:** Paolo Gasparella, Emir Q. Haxhija, Rune Andersen, Maria Barea, Eulalia Baselga, Miguel Bejarano Serrano, Sigurd Berger, Annouk Anne Bisdorff, Olivia Boccara, Petra Borgards, Maria Bom-Sucesso, Laurence M. Boon, Anca Maria Cimpean, Andrea Diociaiuti, Veronika Dvorakova, May El Hachem, Sofia Frisk, Nader Ghaffarpour, Annegret Holm, Alan D. Irvine, Mikkel Kaltoft, Friedrich G. Kapp, Olga Koskova, Kristiina Kyrklund, Miguel Madureira, Darius Palionis, Przemysław Przewratil, Bitten Schönewolf-Greulich, Maria-Corina Stanciulescu, Jaroslav Štěrba, Jukka Tolonen, Birute Vaisnyte, Carine van der Vleuten, Dariusz Wyrzykowski, Leo Schultze Kool, Miikka Vikkula

**Affiliations:** 1 Department of Paediatric and Adolescent Surgery, VASCERN-VASCA European Reference Centre, Medical University of Graz, Auenbruggerplatz 34, 8036 Graz, Austria; 2 Department of Radiology and Nuclear Medicine, VASCERN-VASCA European Reference Centre, Oslo University Hospital, Oslo, Norway; 3 VASCAPA, Vascular Anomaly Patient Association, Brussels, Belgium; 4 Pediatric Dermatology, VASCERN-VASCA European Reference Centre, Hospital Sant Joan de Deu, Barcelona, Spain; 5 VASCERN-VASCA European Reference Centre, AP-HP, Université de Paris, Hôpital Lariboisière, Centre Constitutif Des Malformations Artério Veineuses Superficielles de L’Enfant Et de L’Adulte, NeuroradiologieParis, France; 6 Bundesverband Angeborene Gefäßfehlbildungen E.V., Federal Association of Congenital Vascular Malformation, Mülheim an Der Ruhr, Germany; 7 Pediatric Oncology, VASCERN-VASCA European Reference Centre, Centro Hospitalar Universitário São João, Porto, Portugal; 8 Interventional Radiology, VASCERN-VASCA European Reference Centre, Centro Hospitalar Universitário São João, Porto, Portugal; 9 Center for Vascular Anomalies, Division of Plastic Surgery, VASCERN-VASCA European Reference Centre, University Clinics Saint-Luc, University of Louvain, Brussels, Belgium; 10 Human Molecular Genetics, de Duve Institute, University of Louvain, Ottignies-Louvain-La-Neuve, Belgium; 11 Department of Microscopic Morphology/Histology VASCERN-VASCA European Reference Centre , Victor Babes University of Medicine and Pharmacy, Timișoara, Romania; 12 Dermatology Unit and Genodermatosis Unit, Translational Pediatrics and Clinical Genetics Research Area, VASCERN-VASCA European Reference Centre, Bambino Gesù Children’s Hospital, IRCCS, Rome, Italy; 13 Paediatric Dermatology, Clinical Medicine, VASCERN-VASCA European Reference CentreChildren’s Health IrelandTrinity College Dublin, Crumlin, Ireland; 14 Department of Clinical Genetics, VASCERN-VASCA European Reference Centre, Karolinska University Laboratory, Karolinska University Hospital, Stockholm, Sweden; 15 Department of Reconstructive Plastic and Craniofacial Surgery, VASCERN-VASCA European Reference Centre, Karolinska University Hospital, Stockholm, Sweden; 16 Division of Pediatric Hematology and Oncology, Department of Pediatrics and Adolescent Medicine, Medical Center, Faculty of Medicine, VASCERN-VASCA European Reference CentreUniversity of FreiburgUniversity of Freiburg, Freiburg, Germany; 17 Department of Otorhinolaryngology, Head and Neck Surgery and Audiology, Rigshospitalet, VASCERN-VASCA European Reference Centre, Copenhagen University Hospital, Copenhagen, Denmark; 18 Department of Clinical Genetics, Rigshospitalet, VASCERN-VASCA European Reference Centre, Copenhagen University Hospital, Copenhagen, Denmark; 19 Department of Pediatric Oncology VASCERN-VASCA European Reference Centre, University Hospital Brno, Brno, Czech Republic; 20 Department of Pediatric Surgery, VASCERN-VASCA European Reference Centre, University of Helsinki and Helsinki University Hospital, Helsinki, Finland; 21 Department of Internal Medicine, VASCERN-VASCA European Reference Centre, University of Helsinki and Helsinki University Hospital, Helsinki, Finland; 22 Faculty of Medicine, Vilnius University Hospital Santaros Klinikos VASCERN-VASCA European Reference Centre , Vilnius University, Vilnius, Lithuania; 23 Department of Pediatric Surgery and Oncology, Medical University of Lodz, Łódź, Poland; 24 Department of Dermatology, Radboudumc Expertise Center for Haemangiomas and Congenital Vascular Malformations Nijmegen (Hecovan), Radboud University Medical Center, The Netherlands; VASCERN-VASCA European Reference Centre, Nijmegen, Netherlands; 25 Department of Radiology, VASCERN-VASCA European Reference Centre, Radboudumc Expertise Center for Haemangiomas and Congenital Vascular Malformations Nijmegen (Hecovan), Radboud University Medical Center, Nijmegen, The Netherlands

**Keywords:** Kaposiform hemangioendothelioma, Kasabach-Merrit phenomenon, Vascular tumour, Diagnosis, Treatment, Algorithm, Sirolimus, Patient pathway

## Abstract

The European Reference Network (ERN) on Rare Multisystemic Vascular Diseases (VASCERN) is dedicated to gathering the best expertise in Europe in order to improve and provide high-level healthcare to all patients with rare vascular diseases and provide accessible cross-border healthcare to patients with rare vascular diseases. Kaposiform Hemangioendothelioma (KHE) is a rare vascular tumour with local aggressiveness, often associated with a life-threatening coagulopathy known as the Kasabach-Merritt phenomenon (KMP). The Vascular Anomalies Working Group (VASCA-WG) developed diagnostic and therapeutic pathways for the management of KHE using the nominal group technique (NGT), a well-established, structured, multi-stage, facilitated group meeting technique used to generate consensus statements. The pathways were developed following two face-to-face meetings and several web meetings to facilitate discussion.

*Conclusion*: VASCA has produced this expert opinion, which reflects the strategies developed by experts and patient representatives on how to approach patients with Kaposiform Hemangiothelioma in a practical manner; here, we present an algorithmic flow chart of this work.

**Table:** 

**What is Known:** • *Kaposiform hemangioendothelioma (KHE) is a rare, locally aggressive vascular tumor, often associated with a life threatening coagulopathy, and can be challenging to manage.*
**What is New:** • *The diagnostic and therapeutic pathway for KHE, developed by a multidisciplinary group of experts (VASCA-VASCERN Group), offers concrete offers concrete help in making decisions about managing this condition.*

**What is Known:**

• *Kaposiform hemangioendothelioma (KHE) is a rare, locally aggressive vascular tumor, often associated with a life threatening coagulopathy, and can be challenging to manage.*

**What is New:**

• *The diagnostic and therapeutic pathway for KHE, developed by a multidisciplinary group of experts (VASCA-VASCERN Group), offers concrete offers concrete help in making decisions about managing this condition.*

## Introduction

Kaposiform hemangioendothelioma (KHE) is a rare vascular tumour characterised by a locally aggressive behaviour. It is extremely rare with an incidence of approximately 0.71/1,000,000 in North America [1]. It typically presents in the first year of life with a predilection for the neck and extremities [1, 2]. In most cases, it is associated with the onset of the Kasabach-Merritt phenomenon (KMP), a coagulation disorder characterised by a potentially life-threatening thrombocytopenia and consumptive coagulopathy [3]. It has often been misdiagnosed as an aggressive infantile haemangioma. It can be mistaken for congenital hemangioma, a kaposiform lymphangiomatosis, or a sarcoma, and in particular for tufted angioma (TA) with which it shares clinical and histopathological features, but TA has a less aggressive clinical behaviour. Historically, the management of this tumour has been frustrating due to the lack of effective treatments; however, therapeutic options have improved over the past decade with the introduction of sirolimus, a mammalian target of rapamycin (mTOR) inhibitor [4]. Due to the rarity of this condition, treatment remains challenging and should be reserved for multidisciplinary centres with expertise in vascular anomalies.

At the European level, selected centres of expertise are brought together in European Reference Networks (ERNs), all with the aim of improving patient care by building on collective knowledge through multidisciplinary collaboration. VASCERN, the European Reference Network on Rare Multisystemic Vascular Diseases, is dedicated to gathering European expertise to help patients with rare vascular diseases (with an estimated number of 1.3 million affected patients). VASCERN is coordinated from Paris, France. There are six separate working groups focusing on (i + ii) arterial connective tissue diseases (affecting major arteries from the aorta to small arteries, HTAD and MSA), (iii) hereditary haemorrhagic telangiectasia (HHT), (iv) primary and paediatric lymphedema (PPL), (v) neurovascular diseases (NEUROVASCA), and (vi) vascular anomalies (VASCA). VASCA currently gathers highly specialised multidisciplinary expert teams from 13 health care providers (HCPs), one affiliated partner from 14 EU member states, and three developing partners, each represented by two leads, as well as a number of European Patient Organizations.

## Patients and methods

VASCA is composed of a multidisciplinary group of experts (dermatologists, geneticists, interventional radiologists, paediatricians, paediatric surgeons, plastic surgeons, vascular surgeons, ENT specialists, internal medicine specialists, paediatric haematologists, and oncologists) and patient representatives. They represent the national HCPs endorsed by their governments as board members of the European Reference Network for Rare Multisystemic Vascular Diseases (VASCERN).

Based on the principle that decisions made by a group of experts are better than those made by individual experts, VASCA decided to develop diagnostic and management pathways for KHE using a nominal group technique (NGT), a well-established, structured, multi-stage, facilitated, group meeting technique used to generate consensus documents [5].

The pathways were generated during two face-to-face meetings in September 2023 and October 2024 to facilitate discussion, during TEAMS meetings in 2024, and by email to avoid the influence of the most authoritative members.

Two facilitators were identified: one to propose initial discussion points and draw the pathway and another to chair the discussion. A paediatric surgeon was chosen within the group of experts as the first facilitator due to his experience in the management of KHE. Further decision points were proposed by the group, and the best choices were discussed within the panel of experts. Conflicting points were further discussed until a conclusion was agreed by the European multidisciplinary team. The chair of the group promoted inputs from all members, summarised opinions and reasons for decisions, and identified common ground. No time limit was set for reaching consensus. After the first meeting, the document was circulated by email in the WG to collect further peer comments. A final face-to-face meeting was organised to validate the pathway.

The pathway represents level V evidence derived from expert consensus.

## Results

**Fig. 1:**
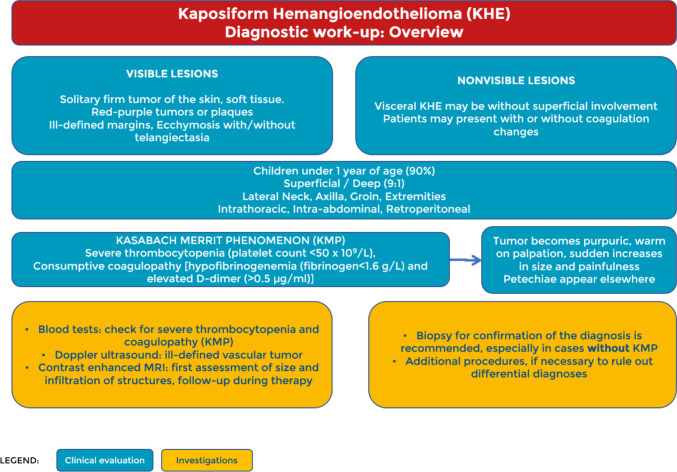
Diagnostic work-up: overview

**Fig. 2:**
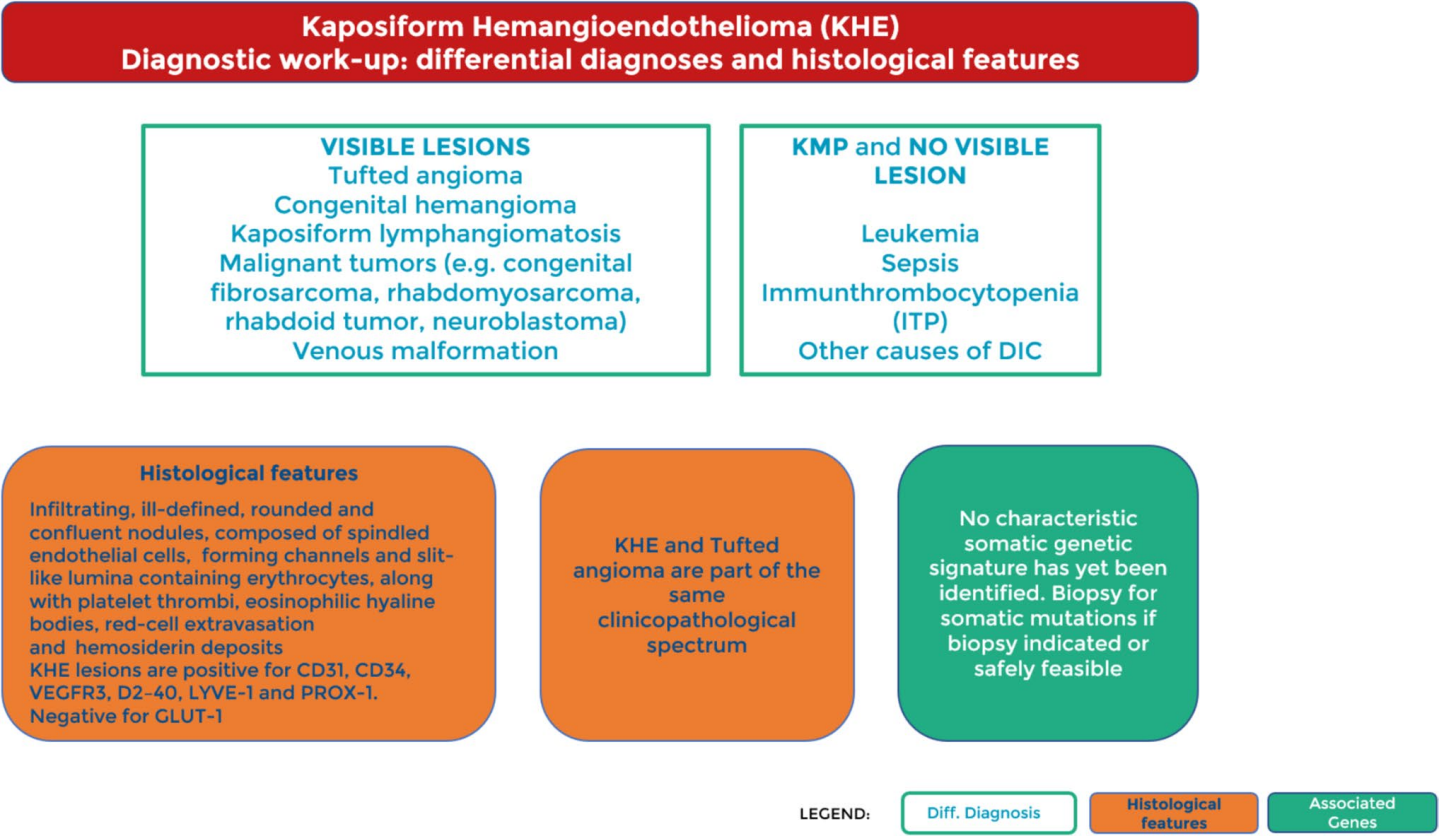
Diagnostic work-up: differential diagnosis and histological features

**Fig. 3:**
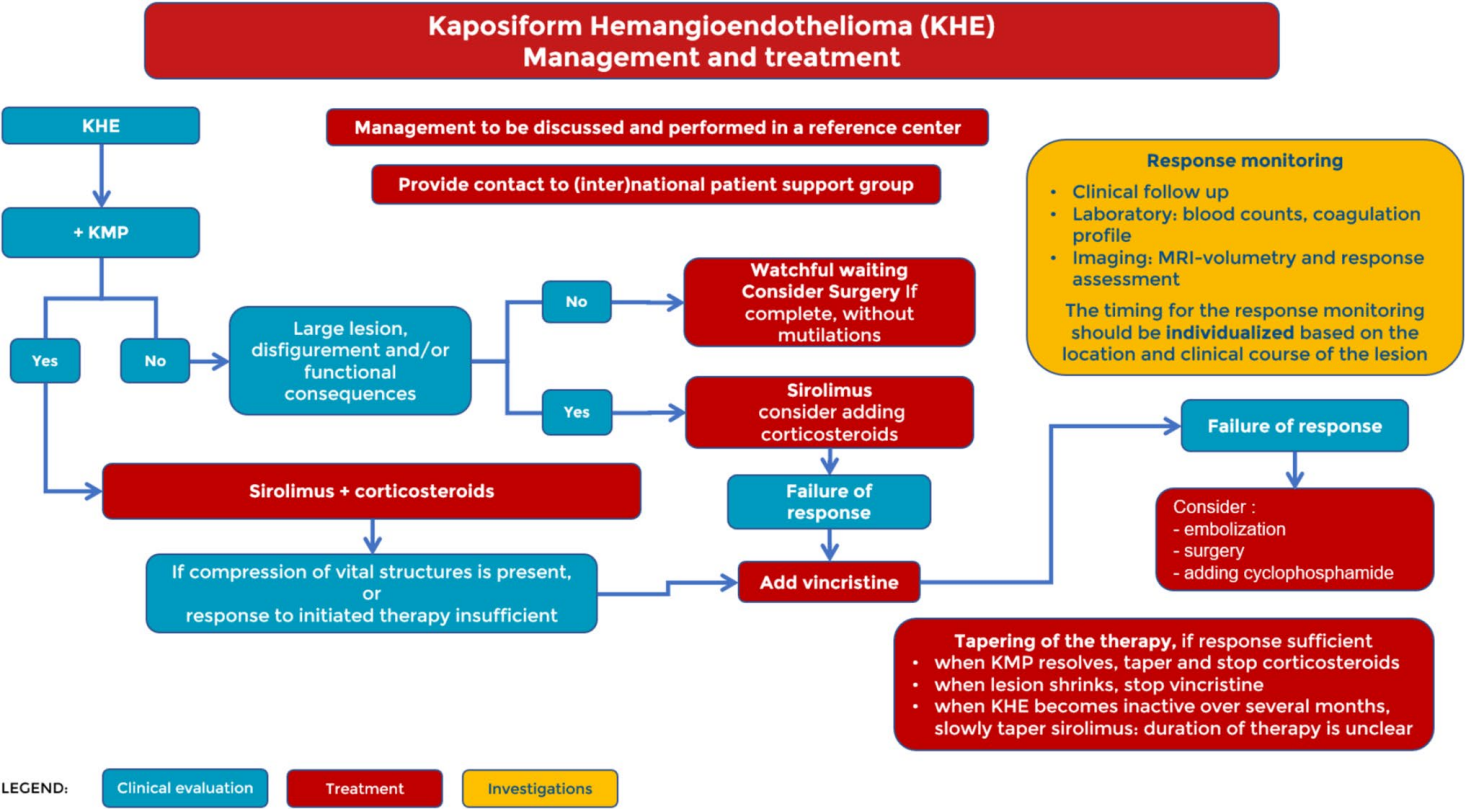
Management and treatment part 1

**Fig. 4:**
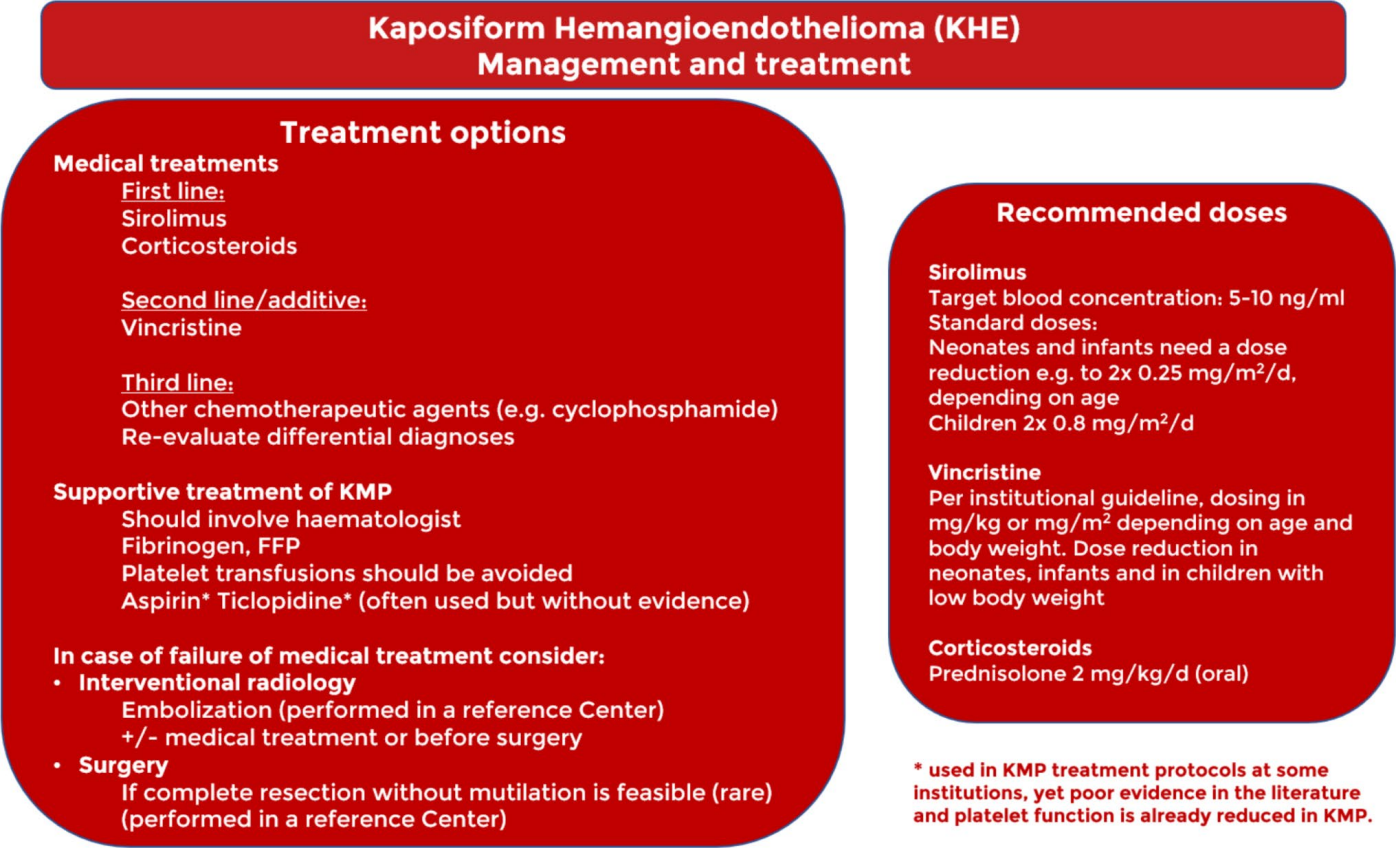
Management and treatment part 2

**Fig. 5:**
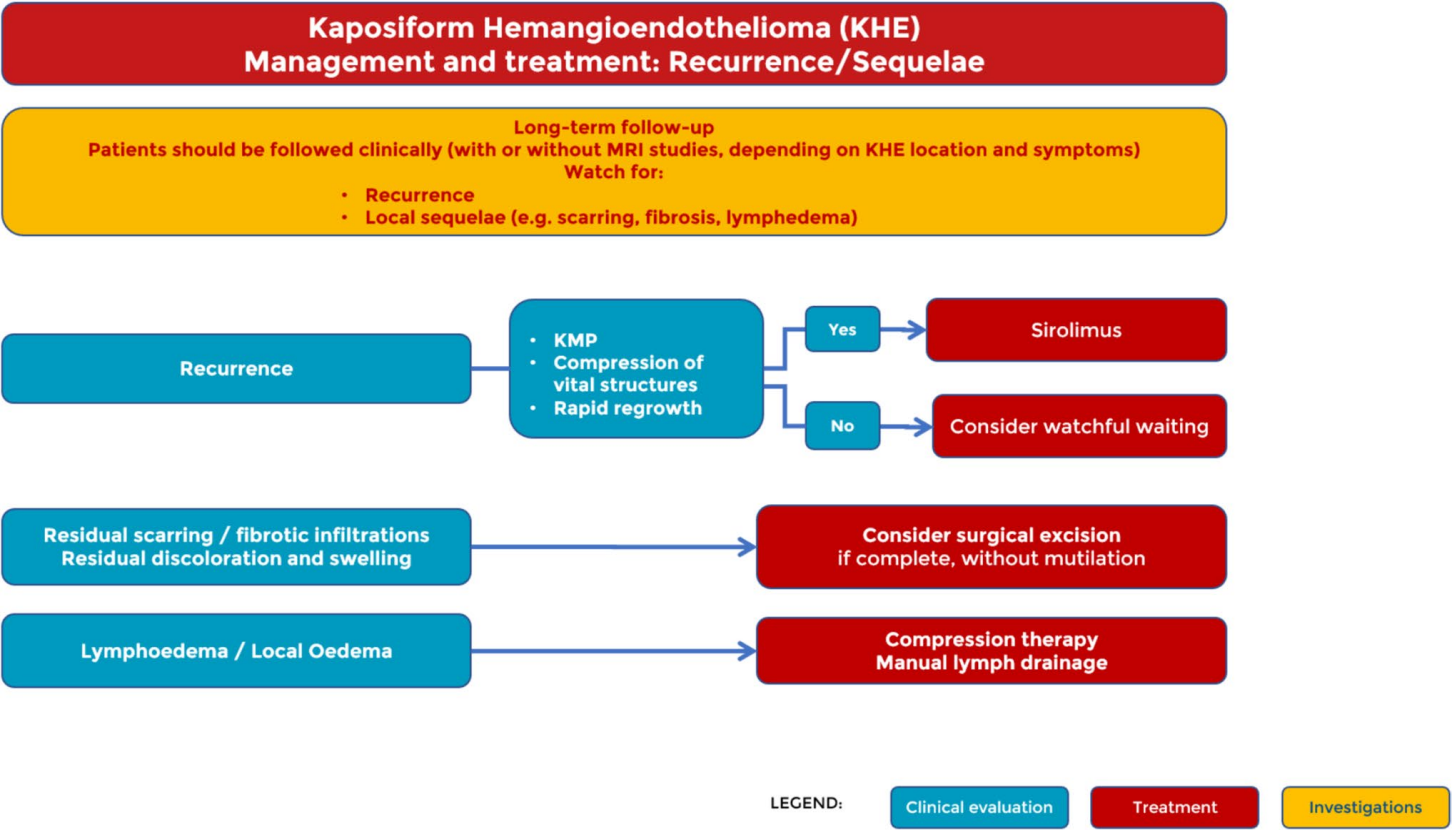
Management and treatment: recurrence/sequelae

The document consists of five pages organised as follows and illustrated in Figs. 1, 2, 3, 4, and 5. The first two pages cover the diagnostic work-up, the third provides a flow chart for management, the fourth gives an overview of current treatment options, and the final page provides suggestions for follow-up.

On the first page of the pathway, we provide an overview of common clinical, laboratory, and imaging features of the diagnosis (Fig. 1).

**Fig. 6:**
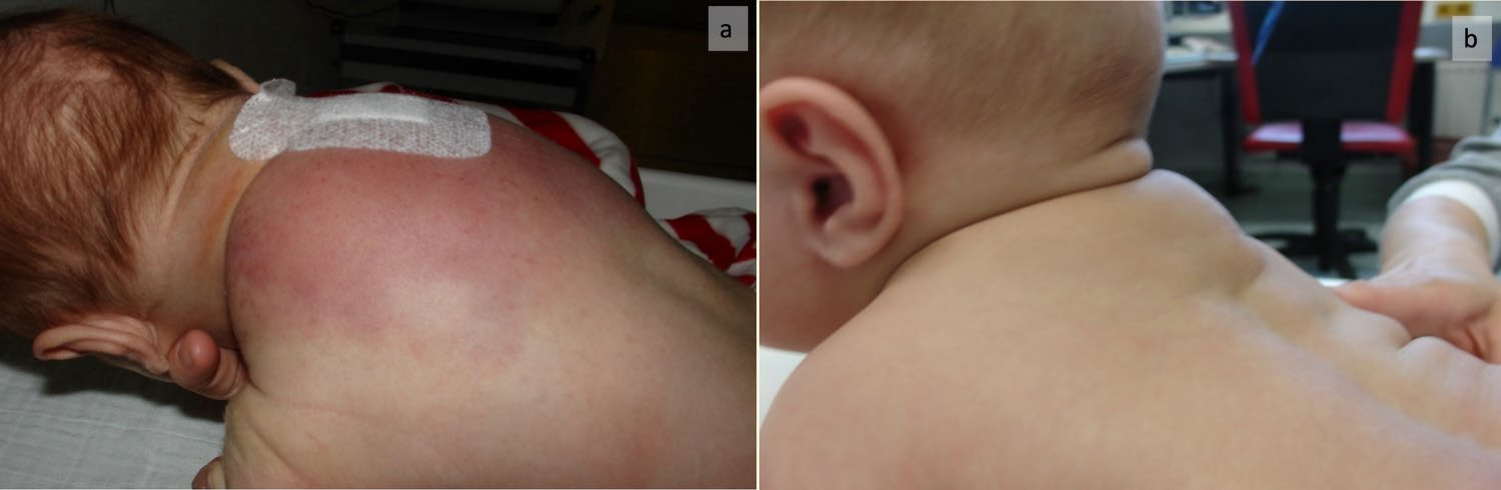
**A** A 1-month-old infant presenting with histopathologically confirmed KHE on the back. **b** The same infant 3 months later, after therapy with sirolimus and corticosteroids

During the diagnostic work-up, lesions can be divided into those that are visible and those that are not. In the former, the onset of a red–purple firm swelling with indeterminate margins in a child suggests the diagnosis (Fig. 6). The identification of visceral lesions without superficial involvement is often challenging and requires advanced imaging. In the case of the Kasabach-Merritt phenomenon (KMP), the onset of typical coagulation changes should suggest the diagnosis [6], but additional diagnostic measures such as diffusion-weighted and dynamic contrast-enhanced MRI sequences are needed to confirm the diagnosis and exclude differential diagnoses. In some cases, the tumour causes symptoms due to the involvement of nearby structures, such as pain and reduced function, respiratory distress, or abdominal distension, but sometimes the diagnosis is made incidentally [2].

KHE shows a characteristic natural history. The tumour appears in most cases before the age of one year [2, 7], and only in 10% of cases does it present with visceral involvement without visible signs on the skin. It can occur in any part of the body, but the most frequently affected regions are the lateral neck, axilla, groin, extremities, thorax, abdominal cavity, and retroperitoneum.

In up to 71% of cases, KHE is associated with KMP [2], a coagulation disorder characterised by severe thrombocytopenia, consumptive coagulopathy, and elevated D-dimers. When KMP occurs, the lesion usually rapidly increases in size and becomes purpuric, warm to palpation, and painful. Furthermore, petechiae may appear at a distance from the lesion, a sign that highlights the severity of the thrombocytopenia [3].

Early in the evaluation of a patient with suspected KHE, laboratory tests, such as platelet count, D-dimers, and fibrinogen, should be performed to assess the presence of coagulation abnormalities due to the high incidence of KMP in patients with KHE.

The first choice of diagnostic imaging is Doppler ultrasound, which shows a hyper-vascularised solid mass with ill-defined margins. This should be followed by contrast-enhanced MRI to define the size and extension of the mass, which will facilitate assessment on follow-up imaging [8]. MRI features include dermal and subcutaneous thickening, infiltrative and ill-defined margins, oedema-like appearance in the surrounding tissue, and dilated fast-flow vessels. Lesions appear iso-hypointense to muscle on T_1_-weighted sequences and hyperintense on T_2_.

It is recommended that the diagnosis be confirmed by histology. Although the group agreed that histopathology should be obtained, it is recognised that in certain patients, the periprocedural risk is too high to obtain a biopsy, and management must be guided by the available diagnostic tools.

Figure 2 emphasises the importance of considering the differential diagnoses of KHE. It is particularly important to rule out malignancies (e.g. congenital fibrosarcoma, rhabdomyosarcoma, rhabdoid tumour, neuroblastoma), in which an incorrect treatment could drastically change the prognosis [9]. KHE lesions are positive for CD31, CD34, ERG, Fli1 (ERGB) and SMA (partially), VEGFR3, D2-40, Lyve1, and Prox1. Negative for GLUT1 and HHV8.

**Fig. 7:**
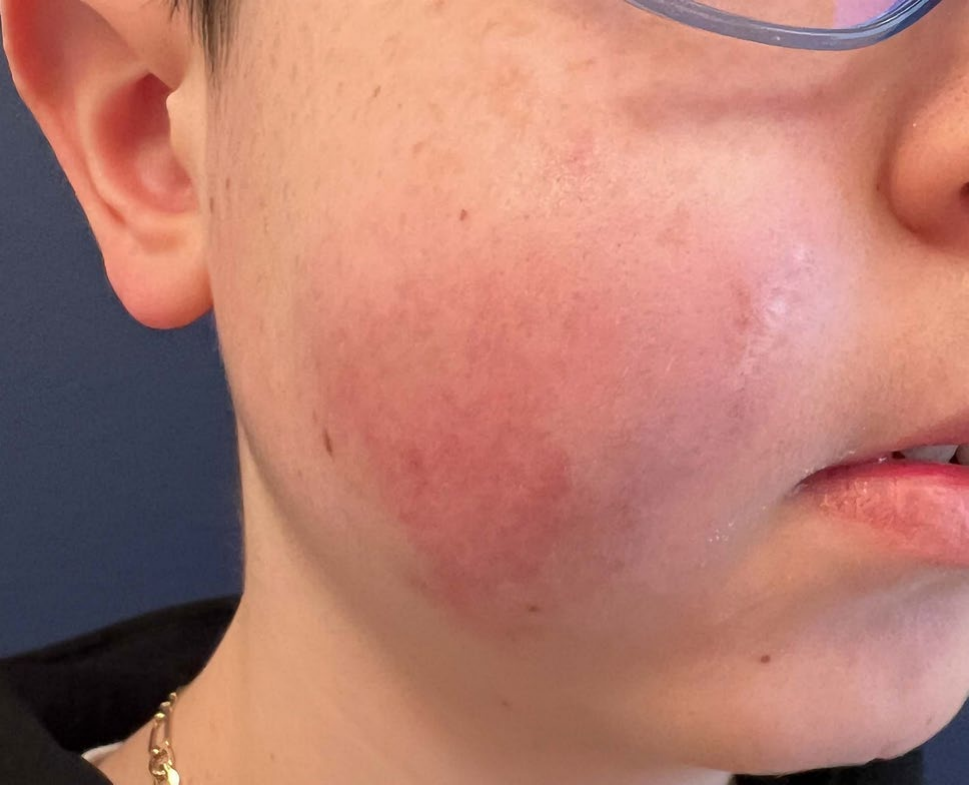
A 10-year-old boy with a histopathologically confirmed tufted angioma (TA) of the right cheek

If the main symptoms are related to coagulation abnormalities such as KMP without a visible lesion, other conditions triggering a disseminated coagulopathy need to be considered, especially in relation to the patient's age. Figure 2 also describes the histological features of KHE. Differentiation from TA can be particularly difficult, as it shares the histopathological features with KHE and represents a less clinically aggressive expression of this continuum (Fig. 7) [10]. Currently, to our knowledge, no pathognomonic genetic variants have been identified in KHE or TA [11]. Molecular profiling is recommended to facilitate precision medicine and future research that may characterise the genetic abnormalities of this tumour.

Once a diagnosis of KHE is suspected, it is important to manage the patient in coordination with a vascular anomaly expert centre. If feasible, the family should be informed about dedicated national and international patient associations, which can help coordinate the social and psychological aspects not directly related to medical treatment.

As mentioned above, an important step is to assess the presence of KMP. If KMP is ruled out, watchful waiting may be considered for small lesions. In these rare cases, radical surgery that avoids functional defects and mutilation can be curative [7]. However, if the tumour is large, disfiguring, and/or has functional or life-threatening consequences, sirolimus therapy should be considered first-line treatment [12]. In case of failure to respond, a re-evaluation of the diagnosis is recommended, and after confirmation, the addition of vincristine can induce a reduction in the size of the mass.

In the presence of KMP, starting with sirolimus [13] and corticosteroids appears to be the best option. A randomised trial of 75 patients with KHE and KMP treated with higher sirolimus trough levels (10–15 ng/mL) demonstrated better outcomes when oral corticosteroids were administered for several weeks, compared with sirolimus alone [14].

In the presence of lesions causing compression of vital structures, or if the response to the initiated therapy appears inadequate, it is advisable to add vincristine to the therapeutic protocol.

In the case of treatment failure, other options such as embolisation [15], surgery, or cyclophosphamide [16] may be considered after reassessing differential diagnoses.

The optimal moment for discontinuing medical treatment remains unclear but should be guided by the clinical response, safety, and tolerability of the medication. Once KMP has resolved and the patient is in a stable clinical condition, it is recommended to taper and discontinue corticosteroid therapy in order to limit corticosteroid exposure at this young age. If Vincristine has been administered, it may be discontinued should the lesion demonstrate no further decrease in size. The duration of sirolimus treatment and how this may affect relapse is a topic of debate. However, when KHE becomes inactive over several months, a slow taper to a very low dose is recommended.

The introduction of sirolimus to the treatment regimen for KHE has significantly improved the prognosis for these patients and has been shown to be efficacious in limiting KMP and consequently reducing mortality [17]. According to recent publications, the combination of sirolimus and corticosteroids as the first-line treatment has been shown to provide stable resolution of coagulation abnormalities [12–14]. Although there are studies reporting the efficacy of sirolimus in infants with lower ranges (2–5 ng/mL) [18], the group agreed that the target blood concentration range at trough levels should be between 5 and 10 ng/mL, with both higher and lower doses being reported as effective in the literature. Notably, caution must be exercised in newborns and infants, as a lower dose is sufficient to achieve these serum concentrations [18, 19]. The risk of infections should be taken into account, and individualised preventive measures such as antibiotic prophylaxis could be considered. In cases where there is a life-threatening localisation or failure to respond to sirolimus and corticosteroids, vincristine should be added to the therapeutic regimen. It is important to note that vincristine requires central venous access [12].

In the event of medical treatment failing, embolisation and surgery are considered valuable options. Embolisation of KHE lesions can be a particularly challenging procedure as it involves cannulation of small vessels in young paediatric patients. It is recommended that this procedure be performed by referral centres. In selected cases, combining the procedure with medical therapy can stabilise the patient. Furthermore, pre-surgical embolisation of the tumour may result in a temporary reduction in volume and reduced intraoperative bleeding. The surgical option should be reserved for rare cases in which a complete excision is possible without mutilation. The procedure should be performed by experienced surgeons, and the decision should be made within a multidisciplinary team, after evaluating all possible options [7].

### Supportive care

KMP is a life-threatening condition, and its treatment must be coordinated by an expert haematologist able to recognise the progression of the disease and adapt therapy accordingly (e.g. administration of fresh frozen plasma (FFP) or fibrinogen). Due to their rapid inactivation and entrapment by the tumour mass, platelet transfusions should be avoided as they cause an increase in tumour mass and worsen thrombocytopenia. Despite the lack of evidence in the literature and the fact that platelet function is already impaired in KMP, some centres include aspirin and/or ticlopidine in their treatment protocols [20].

It is essential to closely monitor the patient's response to treatment, which should include clinical follow-ups, laboratory workups (complete blood count, coagulation profile), and radiological imaging using MRI. Due to variability in the location and clinical course of the lesion, individualisation of the timing of monitoring is appropriate.

We recommend that patients with KHE receive long-term follow-up, focusing on the detection of relapses and management of sequelae, as both are common in this patient population [21]. MRI is the most accurate method, but timing is highly dependent on the location of the tumour, its extension into vital structures, and associated symptoms.

In the case of recurrence, the therapeutic approach is influenced by the presence of KMP, compression of vital structures, or rapid growth of the lesion. In these patients, it is advisable to restart therapy with sirolimus. In all other patients, however, it is reasonable to consider a watchful waiting approach.

In cases of residual local scarring, fibrotic infiltration, redundant skin, residual dyschromia, and residual nodules, surgery can be considered. Residual lymphoedema or local oedema may be present after regression of KHE affecting the extremity. Initiation of compression therapy or manual lymphatic drainage can reduce swelling (22).

## Discussion

In the absence of clinical trials and meta-analyses in the field of rare diseases, expert opinion remains an appropriate tool to improve the quality of care. For rare diseases, level V evidence remains a necessary tool for clinical decision-making. The assignment of the level of evidence does not take into account the value of the processes used to reach an expert opinion.

The quality of an expert panel’s opinion depends on the experience of its members. The expertise in the field of vascular anomalies of VASCA is suggested by the selection of national reference centres, endorsed by their governments and selected by the European Community's ERN network, based on well-defined criteria, including the publication record of the group members in the field of vascular anomalies.

The NGT has been defined by Ven and Delbecq as “a structured meeting which seeks to provide an orderly procedure for obtaining qualitative information from target groups who are most closely associated with a problem area” (Ven and Delbecq, 1974). The structured process allows the participants to decide which issues require further discussion, thereby avoiding the domination of the debate by more authoritative or dominant members. Moreover, the facilitator ensures equal participation of all group members in conflicting concepts. A limitation of the process is the lack of anonymity guaranteed by the Delphi method, and therefore the inability to completely avoid the authority and personality of some experts driving the process.

Using NGT, VASCA proposes an expert opinion on the current optimal approach to patients with KHE as a useful tool to improve the care and management of these patients. Further clinical studies will contribute to future updates of this algorithm.

## Data Availability

No datasets were generated or analysed during the current study.
